# Cheesecake Customized Using Juice and By-Products from Prickly Pears: A Case Study of Recycling and Environmental Impact Evaluation

**DOI:** 10.3390/foods14071159

**Published:** 2025-03-26

**Authors:** Alessia Le Rose, Olimpia Panza, Dario Caro, Amalia Conte, Matteo Alessandro Del Nobile

**Affiliations:** 1 Ecodynamics Group, Department of Physical Sciences, Earth, Environment, University of Siena, Piazzetta Enzo Tiezzi, 1, 53100 Siena, Italy; alessia.lerose@student.unisi.it (A.L.R.); caro2@unisi.it (D.C.); 2 Department of Science, Technology and Society, University School for Advanced Studies IUSS Pavia, 27100 Pavia, Italy; 3 Department of Humanistic Studies, Letters, Cultural Heritage, Educational Sciences, University of Foggia, Via Arpi, 71121 Foggia, Italy; olimpia.panza@unifg.it; 4 Department of Economics, Management and Territory, University of Foggia, Via A. da Zara, 71122 Foggia, Italy; matteo.delnobile@unifg.it

**Keywords:** cheesecake, prickly pears, by-product valorization, recycling, environmental impact, sustainable food

## Abstract

Due to the increasing concern about the negative impact of the modern food system and the need to design foods to improve their healthiness and sustainability, in the current study, a fortified cheesecake was developed by using juice, peels, and pomace from prickly pears, which are fruit by-products rich in active compounds. After proper dehydration and being ground to produce a fine powder, some traditional ingredients were substituted with fruit juice and by-products. The water content loss during dehydration and the energy consumed per g of dehydrated by-product were assessed using a proper mathematical approach. A sensory evaluation was carried out using a panel test, thus verifying that the new dessert made with prickly pears was comparable to the traditional one; both recorded high scores of acceptability (sensory score ranged between 8 and 9). The centesimal composition of the two cheesecakes also demonstrated that the ingredient substitution did not affect the energetic value of the final product (290 vs. 248 kcal/100 g); on the contrary, it promoted an increase in carbohydrates (27.38 vs. 26.26 g/100 g), lipids (16.98 vs. 12.94 g/100 g), and total fibers (5.7 vs. 4.2 g/100 g). To demonstrate that the recycling of by-products from prickly pears could represent an advantage from an environmental point of view, a full Life Cycle Assessment (LCA) was carried out. In relation to this, three environmental impact categories, such as Global Warming Potential, Acidification and Eutrophication, which are associated with three different biowaste treatment options—such as composting, landfilling, and recycling—were assessed. The results from the LCA highlighted that recycling always emerged as the most sustainable biowaste management option. For all environmental impact categories analyzed, recycling resulted in an overall environmental saving (−7.63 kgCO_2_eq/kg biowaste; −0.116 kgSO_2_eq/kg biowaste; and −0.055 kgPO_4_^3−^eq/kg biowaste). In addition, the comparison between the traditional cheesecake and the fortified one, in terms of impacts per kg of cheesecake, demonstrated that replacing food items with recycled biowaste may result in a general reduction in emissions and resources. Therefore, this case study represents a valid example of zero-waste production, offering a concrete suggestion as to how processed foods can be redesigned to make them healthier from a more sustainable perspective.

## 1. Introduction

Recently, there has been a growing interest in customized food with a low environmental impact [1,2,3]. In this context a lot of research can be found dealing with horticultural by-products recycled for food fortification [4,5,6]. By-products are rich in bioactive compounds [7], and their recycling in food science is generally intended as a new direction to manage food loss and mitigate climate change [8,9]. Among by-products, peels from prickly pears play an important role, being a source of dietary fibers, proteins, and antioxidant compounds [10,11]. Prickly pear cactus is a tropical or subtropical plant that is mostly derived from Mexico but can also be found throughout the Mediterranean basin, with a world production of about 1 million tons per year. The fruit is widely used as a fresh fruit or for manufacturing fruit juice [12] and alcoholic beverages [13]. Fruit peels, which represent a major form of waste, are rich in polyphenolic compounds; their health benefits as functional agents indicate that they have a high potential for use as food ingredients. Chougui et al. [14] studied the application of the hydro-ethanolic extract of prickly pear peel to preserve margarine. Bouazizi et al. [15] and Parafati et al. [16] have used prickly pear peel powder as a functional ingredient to formulate bread and biscuits. Di Lucia et al. [17] applied entire prickly pears to improve the storability of cod burgers. Despite these valid applications of recycled by-products in the food sector, a knowledge gap remains in relation to a concrete evaluation of the environmental benefits associated with their recycling.

Quantifying environmental impact is difficult; thus, the available evidence on sustainable food optimized with the addition of by-products is rather sparse and inconclusive [18]. Producing personalized products with sustainable characteristics requires not only specialized skills for product designing and manufacturing but also time for environmental impact evaluation, which is challenging when compared to mass-produced foods. A focal point of Directive 2008/98/EC of the European Parliament and Council concerns the management of the large volumes of biowaste that are produced in the EU [19], which can lead to significant environmental and economic risks [20]. In this context, the Directive emphasizes the key role of recycling in the context of a circular economy [21]. Although the recycling of biowaste may transform biowaste into value-added products in line with a circular economy perspective [22,23,24], it also requires energy and, sometimes, can be in competition with other traditional biowaste management options.

Landfilling is generally considered to be the most viable economic management of biowaste, but it has a significant environmental impact, including the production of methane from the decomposition of biodegradable materials [25]. The composting of biowaste returns nutrients to the soil, enhancing its quality while reducing the need for chemical fertilizers and water [26]; however, it is a source of ammonia that causes acidification [27]. Therefore, it becomes necessary to assess the environmental burden of different biowaste management options. Sustainability and food customization are relevant to each other as consumer demands for healthier products increase and governments introduce new legislation to help mitigate the negative impact of foods on human and environmental health [2]. In this context, assessing the environmental impact of sustainable foods that are optimized with by-products by using standardized methodology represents a crucial prerequisite to establish their effective environmental performances and lead policymakers and stakeholders.

The current study adds new insight to the existing body of literature because it represents a first attempt to measure the real advantages relating to the environmental impact of a fortified cheesecake, which has been customized using both the juice and by-products (pomace and peels) of prickly pear processing, taking into consideration that by-product composition may promote nutritional enhancement, texture stability, and more prized flavors in the fortified dessert formulation.

Cheesecake is a popular dessert made up of biscuits, pastry or sponge cake, and cheese. There are numerous cheesecake recipes that use many types of cheese throughout the world [28], which significantly affect sensorial properties, primarily texture, taste, and appearance [29]. Desserts are typical products that require personalization, because of the rapid rise in blood sugar they cause when eating, as well as their high fat concentration [30]. Park et al. [31] provided guidance on variables that can be considered for adding functional materials in relation to the manufacture of a functional 3D-printed cheesecake. These authors studied the rheological, textural, and functional characteristics of dessert containing guava leaf, green tea, and barley sprout powders. Abdel-Salam and Ahmed [32] successfully developed a functional cheesecake for diabetics, where the ingredients of the regular cheesecake were replaced by those with a lower caloric value. Gutierrez et al. [33] proposed a study on the environmental impact of a packaged cheesecake. This work aimed to identify improvements in food packaging solutions that are able to minimize environmental externalities while maximizing economic sustainability. To this end, the environmental impact of packaging and food losses, as well as the balance between the two, were examined in relation to a cheesecake packaged in low-density polyethylene film and in barrier systems under modified atmospheric conditions (MAPs). The results of the study of Gutierrez et al. [33] show that MAPs could considerably extend the shelf life of cheesecakes, thereby reducing food waste and decreasing the overall environmental impact.

In this study, the effects of prickly pears added to cheesecake were studied from a sensory point of view, evaluating the odor, color, texture, and overall quality of both a control and a fortified cheesecake. The chemical composition was also assessed in terms of lipids, carbohydrates, dietary fiber content, proteins, moisture, sodium, sodium chloride, ash, and total energy value in order to carefully consider the nutritional impact of switching from one type of product to another, as well as demonstrating that a real nutritional advantage was gained in substituting the traditional ingredients with new ones. By developing a full Life Cycle Assessment (LCA), the first objective was to estimate three significant environmental categories, i.e., Global Warming Potential, Acidification, and Eutrophication, which are associated with three different biowaste treatment options—composting, landfilling, and recycling. The second objective was to compare the traditional cheesecake with the “fortified cheesecake” to assess the potential environmental benefits.

## 2. Materials and Methods

### 2.1. Prickly Pear Processing and By-Product Dehydration

A local dealer (Manfredonia, Puglia, Italy) kindly provided red prickly pears (*Opuntia ficus-indica* L. Mill.), cultivar Sanguigna, which were stored at 4 °C prior to processing. Then, they were carefully washed, as reported by Panza et al. [34]. After cleaning, the peel was manually separated from the pulp. Subsequently, the pulp was centrifuged using a device (Slow Juicer H320N) manufactured by Huron (Verona, Italy), obtaining prickly pear juice and pulp waste, which is labeled as prickly pear pomace. The juice was stored at −18 °C until use, while the pomace and fruit peel were dried at 60 °C in a conventional dryer (PF–SIC CO80PRO, SICCOTECH, Campobasso, Italy), with the relative humidity set to 5%. A hot air-drying process with natural convection at room pressure was used. The dryer cabinet had a volume of 0.6 m^3^ and contained 20 racks. For dehydration, about 1 kg of fresh by-products was distributed over different racks of the cabinet to form a uniform and thin layer. After dehydration, both by-products were milled to obtain a fine powder (˂500 µm), before being stored at 4 °C under vacuum conditions.

### 2.2. Cheesecake Production

Two types of cheesecake were prepared—a control cheesecake and a cheesecake fortified with prickly pears (including juice and by-products). The two recipes are reported in Table 1. Both dessert types comprised three parts—a base, a filling, and a topping. While the base was the same for both samples, the filling and topping differed. The base was made by mixing 50 g of butter with 115 g of biscuit crumbs; the mixture was pressed evenly into molds and refrigerated at 4 °C for approximately 20 min. The control filling was prepared by mixing 175 g of cheese, 100 g of sugar, and 100 g of yogurt, before adding 100 g of fresh cream, which had previously been whipped. The filling was also stored at 4 °C. Subsequently, 40 g of solution (water/milk; 50:50) was thickened with food-grade gelatin and was mixed with the previously prepared filling. For the modified filling, part of the fresh cream was replaced with prickly pear peel and pomace powders, while both water and milk were completely replaced with prickly pear juice (Table 1). For the control topping, jam (125 g) was thickened with pectin (2 g), which had previously been dissolved in water (15 g), by boiling and cooling in the fridge at 4 °C. For the modified topping, part of the jam was replaced with prickly pear peel and pomace powders, while the water was replaced with prickly pear juice (Table 1). The amount of prickly pear peel and pomace powders was precisely calculated according to the amount of fruit juice to be used in the formulation, according to a zero-waste approach. It was previously calculated that 150 g of juice generally generates 33.37 g of dehydrated peel powder and 36.28 g of dehydrated pomace powder. The amounts of these two ingredients from the by-products were precisely divided between the filling and the topping in order to respect the above amounts. The optimization of the amounts for the fortified formulation was carried out through several preliminary experimental tests conducted in our lab, whose data were not published. When the base, filling, and topping of both cheesecakes were ready to be used, the two desserts were prepared by spreading each filling on the base; after 1 h of refrigeration, the relative control and fortified topping were added on the top of each dessert. The two cheesecakes were stored at 4 °C before further analyses.

### 2.3. Cheesecake Centesimal Composition

On both cheesecake samples, lipids, carbohydrates, dietary fiber content, proteins, moisture, sodium, sodium chloride, ash, and total energy value were measured. The analyses were performed using NIRO SRL laboratory (CB, Campobasso, Italy). The total metabolizable energy was determined according to Reg. UE 1169/2011 (25 October 2011), expressed in kilocalories (kcal/100 g). The lipid content was measured according to ISTISAN 1996/34, expressed in g/100 g [35]. The total carbohydrates were calculated according to Reg. UE 1169/2011 (25 October 2011), expressed in g/100 g. The dietary fiber content was measured according to AOAC Official Method [36], expressed in g/100 g. The protein content was determined according to ISO 1871:2009, expressed in g/100 g [37]. The moisture content was determined according to ISTISAN 1996/34 [38], expressed in percentage value. The ash content was determined according to UNI EN ISO 2171:2023, expressed in g/100 g [39].

### 2.4. Cheesecake Sensory Evaluation

Sensory analysis aimed to assess the acceptability of both the control and the fortified cheesecake. As such, sensory evaluation was carried out by seven trained panel members. The panelists have several years of food product sensory evaluation experience; however, before the current test, they received two training sessions carried out over 2 days (1 h/session), using traditional cheesecake to select the adequate sensory parameters, to identify the proper scale to be used, and to align their judgements. During the panel test, both control and fortified cheesecake samples were presented to each panelist in a random order at room temperature. The samples were evaluated for the texture, flavor, color, and taste of each entire dessert, which was made up of a base, a filling, and a topping (the sensory evaluation form is available in the Supplementary Materials). An overall acceptability was also considered as a form of the general sensory quality of each cheesecake. A 9-point scale was used for the assessment of each specific sensory attribute and for the overall acceptability (1 = lowest score; 9 = highest score). A score equal to 5 was taken as the threshold for acceptability. An appropriate protocol for protecting the rights and privacy of all participants was utilized during the execution of the research, considering the verbal consent of the participants, no coercion to participate, the ability to withdraw from the study at any time, the full disclosure of study requirements and risks, and not releasing participant data without their knowledge. Experimental data from sensory evaluation were compared using one-way ANOVA analysis. To determine significant differences among samples, Duncan’s multiple range test, with the option of homogeneous groups (*p* < 0.05), was carried out using STATISTICA 7.1 for Windows (StatSoft, Inc., Tulsa, OK, USA).

### 2.5. By-Product Water Content

The by-product water content was evaluated according to the following equation:

(1) $$C\left(t\right)=\frac{W\left(t\right)-W_{F}}{W_{F}}\cdot 100$$

where $C\left(t\right)$ is the sample’s water content at time t, expressed as $\left[\frac{g\;\mathrm{water}}{100\;g\;\mathrm{dry}\;\mathrm{matter}}\right]$; $W\left(t\right)$ is the weight of the sample at time t, expressed as $\left[g\right]$; and $W_{F}$ is the weight of the sample after it has been kept at 130 °C until all the water was desorbed, which was expressed as $\left[g\right]$.

The amount of water desorbed at time t $\left(M_{H_{2}O}\left(t\right)\right)$ was calculated according to the following equation:

(2) $$M_{H_{2}O}\left(t\right)=C_{0}-C\left(t\right)$$

where $C_{0}$ is the sample’s initial water content. The amount of water desorbed at equilibrium $\left(M_{H_{2}O}^{\infty }\right)$ was estimated according to the following expression:

(3) $$M_{H_{2}O}^{\infty }=C_{0}-C_{\infty }$$

where $C_{\infty }$ is the sample’s water content at the end of the dehydration process.

### 2.6. Energy Consumption of the By-Product’s Dehydration Process

The amount of energy consumed per g of dehydrated by-product ($\tilde{E}\left(t\right)$) is given by the following expression:

(4) $$\tilde{E}\left(t\right)=\frac{E\left(t\right)}{m_{Tot}\left(t\right)}$$

where $E\left(t\right)$ is the energy consumed by the dehydrator at time t, and $m_{Tot}\left(t\right)$ is the mass of the dehydrated by-product at time t.

It can be easily demonstrated that the mass of the dehydrated by-product ($m_{Tot}\left(t\right)$) is related to $M_{H_{2}O}\left(t\right)$ through the following expression:

(5) $$m_{Tot}\left(t\right)=m_{Tot}^{0}\cdot \left(1-\frac{x_{dm}^{0}}{100}\cdot M_{H_{2}O}\left(t\right)\right)$$

where $m_{Tot}^{0}$ is the initial value of $m_{Tot}\left(t\right)$, and $x_{dm}^{0}$ is the initial value of dry matter mass fraction (i.e., $x_{dm}^{0}=\frac{m_{dm}}{m_{Tot}^{0}}$, where $m_{dm}$ is the food dry matter). In this study, the model proposed by Conte et al. [40] was used to describe the dehydration kinetics of the investigated by-product. The reader is invited to refer to the study of Conte et al. [41] for mechanistic and theoretical reasoning, along with the limitations of the model, which are discussed in detail when the model was presented for the first time. Based on what was reported in the paper of Conte et al. [40], the by-product dehydration kinetics can be described according to the following relationships:

(6) $$\begin{array}{c} 0\leq t\leq t_{c}\\ M_{H_{2}O}\left(t\right)=K_{1}\cdot t \end{array}$$

(7) $$\begin{array}{c} t>t_{c}\\ M_{H_{2}O}\left(t\right)=K_{1}\cdot t_{c}+K_{2}\cdot \left\{1-exp\left[-\left(t-t_{c}\right)\cdot \left(\frac{K_{1}}{K_{2}}\right)\right]\right\} \end{array}$$

where $K_{1}$ is the desorption rate during the 1st Stage $\left[\frac{g\;\mathrm{desorbed}\;\mathrm{water}}{100\;g\;\mathrm{dry}\;\mathrm{matter}}\cdot \frac{1}{\min}\right]$, and $K_{2}$ is the maximum amount of water desorbed during the 2nd Stage $\left[\frac{g\;\mathrm{desorbed}\;\mathrm{water}}{100\;g\;\mathrm{dry}\;\mathrm{matter}}\right]$. The reader is invited to refer to the study of Conte et al. [40] for a detailed description of the model, as well as the hypotheses that were made to derive it.

To describe the energy consumed by the dehydrator, the following expression was used:

(8) $$E\left(t\right)=\gamma \cdot t$$

where γ is the energy power provided to the dehydrator.

The extent of the dehydration process ($ext\%\left(t\right)$) was defined according to the following expression:

(9) $$ext\%\left(t\right)=\frac{M_{H_{2}O}\left(t\right)}{M_{H_{2}O}^{\infty }}\cdot 100$$

If the dehydration kinetics of the investigated by-products follows the model proposed by Conte et al. [40] (i.e., Equations (6) and (7)), it can be demonstrated that the amount of energy consumed per gram of dehydrated food is related to the extent of the dehydration process through the following expressions:

(10) $$\begin{array}{c} 0\leq ext\%\leq \frac{K_{1}\cdot t_{c}}{M_{H_{2}O}^{\infty }}\cdot 100\\ \tilde{E}\left(ext\%\right)=\frac{\gamma \cdot \left(\frac{M_{H_{2}O}^{\infty }}{K_{1}\cdot 100}\cdot ext\%\right)}{m_{Tot}^{0}\cdot \left(1-\frac{x_{dm}^{0}}{100}\cdot ext\%\cdot \frac{M_{H_{2}O}^{\infty }}{100}\right)} \end{array}$$

(11) $$\begin{array}{c} ext\%>\frac{K_{1}\cdot t_{c}}{M_{H_{2}O}^{\infty }}\cdot 100\\ \tilde{E}\left(ext\%\right)=\frac{\gamma \cdot \left[t_{c}-ln\left(\frac{K_{1}\cdot t_{c}+K_{2}-ext\%\cdot \frac{M_{H_{2}O}^{\infty }}{100}}{K_{2}}\right)\cdot \frac{K_{2}}{K_{1}}\right]}{m_{Tot}^{0}\cdot \left(1-\frac{x_{dm}^{0}}{100}\cdot ext\%\cdot \frac{M_{H_{2}O}^{\infty }}{100}\right)} \end{array}$$

### 2.7. Life Cycle Assessment (LCA) of Cheesecakes

**Figure 1:**
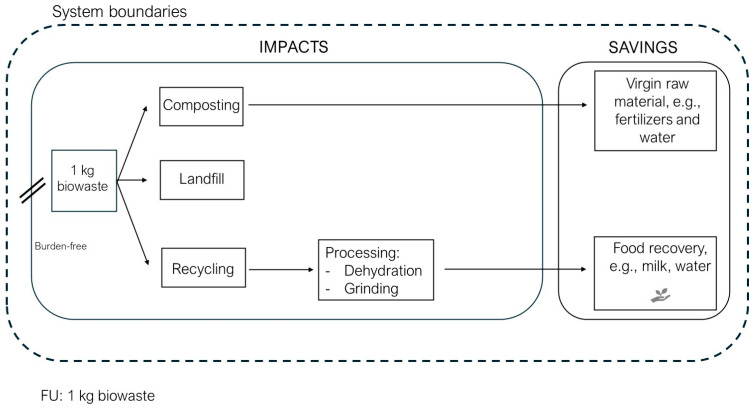
System boundaries and functional unit for the first objective of the paper.

LCAs are a methodology used to evaluate the environmental impacts and benefits associated with a product or service throughout its entire life cycle, from raw material extraction to the disposal phase [42]. At the international level, it is regulated by the ISO 14040 series of standards, according to which an LCA study is structured into the following four phases: goal and scope definition, inventory analysis, impact assessment, and interpretation of results [43,44]. Based on the two objectives of this study, the prickly pear by-products were considered as generic biowaste. The activities and their corresponding environmental impacts prior to biowaste generation were excluded from the analysis, as is common in most LCA studies on waste management [45]. Therefore, all the management options considered in this study are intended to be “burden-free” (Figure 1).

The functional unit defines the specific functions of the product system and provides a reference to which all its inputs and outputs can be correlated [46]. The functional unit (FU) was defined as 1 kg of biowaste. Transportation was excluded from all the scenarios analyzed, as it is assumed to be comparable among the three scenarios analyzed. For the second objective of the study, the entire supply chain of all the ingredients in the cheesecake was assessed. The environmental impacts of the “biscuit” and “jam” ingredients were, respectively, extracted from the Environmental Product Declaration (EPD) of Pavesi Biscuits [47] and from the study of Gallo et al. [48]. Furthermore, since the databases used did not include the ingredient ‘prickly pear juice’, pomegranate cultivation was chosen as a reference to replace prickly pear, as both are tropical fruits [49] that grow under similar climatic conditions [50]. For the industrial production of juice, data and associated impacts were extracted from the EPD related to apple juice produced in Italy [51]. Finally, the food additives used in the production of the cheesecake (pectin and isinglass) were excluded from the analysis due to a lack of data. However, they represent just 0.79% of the total weight of the cheesecake; thus, their contribution can be considered negligible.

#### 2.7.1. Life Cycle Inventory (LCI)

For the first objective of the study, primary data, such as direct data provided by the laboratory of the University of Foggia, were used to analyze the recycling process, e.g., the energy consumption as a result of the dehydration and grinding of the prickly pears. For composting and landfilling, input data from the selected databases were used [52] (Table S1, Supplementary Materials) except for those related to the benefits of compost application, which were extrapolated from the work of Saer et al. [27]. Saer et al.’s study reported an environmental saving, in terms of Global Warming Potential (GWP), of 0.675 t CO_2_ eq per ton of compost, thus assuming a conversion factor of 0.335. For the second objective, secondary data from different sources and databases were used for each ingredient of the cheesecake [53] (Table S2, Supplementary Materials).

#### 2.7.2. Life Cycle Impact Assessment (LCIA)

The analysis was conducted using the “CML-IA baseline v 3.06” methodology of the SimaPro v. 9.6.0.1 software to assess the potential contributions to each impact category. In particular, the environmental indicators and the impact categories selected for both aims were Global Warming Potential (GWP100; expressed in kgCO_2_eq), Acidification Potential (AP; expressed in kgSO_2_eq), and Eutrophication Potential (EP; expressed in kgPO_4_^3−^eq). The selection of these impact categories is based on the direct influence that the analyzed systems have on them. For instance, energy consumption in the recycling process is associated with the use of fossil fuels, such as coal, oil, and natural gas, which significantly contribute to GWP [54]. Similarly, for the same impact category, methane (CH_4_) and nitrous oxide (N_2_O) emissions from landfilling are relevant [27,55]. The Acidification Potential is affected by composting [56,57] releasing ammonia. The Eutrophication Potential was selected due to the high moisture content of food waste, which promotes the generation of nutrient-rich leachate during degradation processes [58]. For the second objective, the same impact categories were analyzed, in line with other studies that focus on the environmental impact assessment of specific food categories [59] or on biowaste recycling [60].

## 3. Results and Discussion

### 3.1. Cheesecake Composition and Sensory Acceptability

Table 2 reports results recorded in the centesimal composition of both cheesecakes. As can be seen, the fortification of cheesecake with prickly pear juice and by-products allowed for the production of a dessert that is richer than the control one, in terms of carbohydrates, lipids, and fibers. By comparing the two products, an increase in the total energetic value was also found (248 vs. 290 kcal/100 g). This experimental evidence is in line with other findings in the literature where prickly pear by-products were used as functional ingredients [15,16].

**Figure 2:**
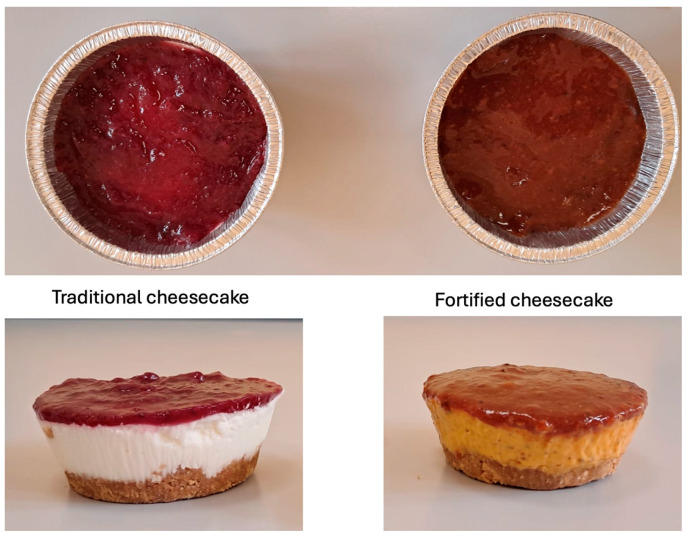
Picture of both the fortified and the control cheesecake.

The two cheesecakes were assessed from a sensory point of view by expert judges. A picture of both cheesecakes is shown in Figure 2. In the picture, there is an image of the topping and also of the entire product. The main difference is in the color of filling, which changes from white in the control to orange in the fortified dessert. The topping is brilliant purple in the control and a dark orange–yellow in the fortified product. It can be inferred that both samples retain the typical fruit color.

**Figure 3:**
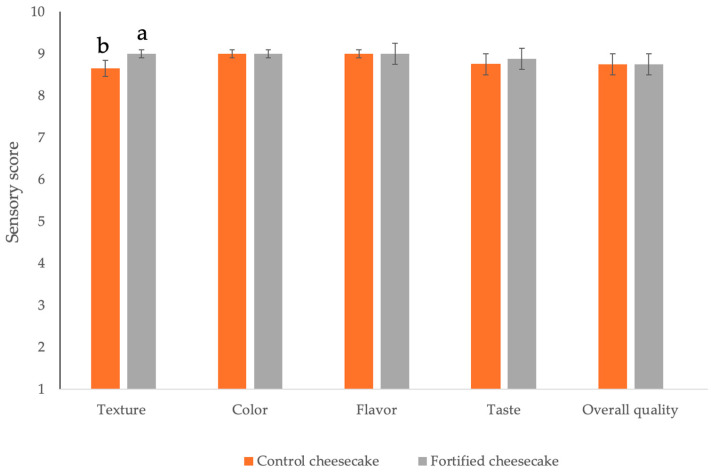
Sensory evaluation of both cheesecakes. Data are the mean score of each parameter, while error bars indicate standard deviation. Different letters (a, b) for texture parameter indicate statistically significant differences in the data (*p* < 0.05).

Figure 3 shows results from the panel test. As can be seen, both types of dessert were appreciated by the panelists, who scored all the parameters between 8 and 9; these scores are considered to be at the top of the acceptability scale. It is worth noting that apart from a slight difference in terms of texture, which was judged to be better in the fortified cheesecake, the two desserts recorded sensory score values without any statistically significant differences (*p* > 0.05) between them. These results demonstrate that the adoption of new ingredients recycled from prickly pears, despite the nutritional differences between the two cheesecakes, did not compromise the final product and no defects were perceived during the evaluation [17]. Contrary to what one would expect, the addition of prickly pears promoted an improvement in sensory quality, most probably because the higher lipid content may influence the creaminess and mouthfeel, thus improving product’s flavor and taste; additionally, the higher carbohydrate and fiber content may influence the texture [61]. According to the single scores received from each parameter, it was verified that no statistically significant differences (*p* > 0.05) were found in terms of overall acceptability. Both desserts recorded a high score of overall acceptability.

### 3.2. Energy Consumption of the By-Product Dehydration Process

**Figure 4:**
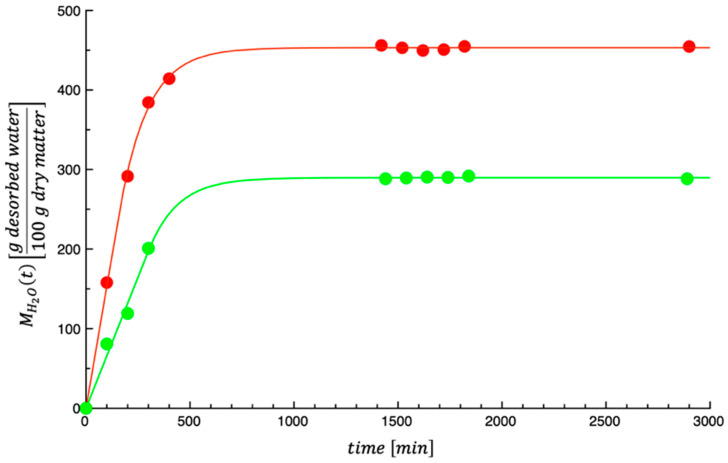
Dehydration kinetics of the investigated prickly pear by-products. Green color = prickly pear pomace; red color = prickly pear peels.

Figure 4 shows the dehydration kinetics of the investigated by-products. The curves shown in the figure are the best fit of the model proposed by Conte et al. [40], i.e., Equations (6) and (7), to the dehydration kinetic data.

The mean relative deviation modulus $\left(\bar{E}\%\right)$ was used to measure the goodness of fit [62]:

(12) $$\bar{E}\%=\frac{100}{N}\cdot \sum_{i=1}^{i=N}\frac{\left|M_{i}^{exp}-M_{i}^{pred}\right|}{M_{i}^{exp}}$$

where N is the number of experimental data, $M_{i}^{exp}$ is the experimental value, and $M_{i}^{pred}$ is the predicted value.

The calculated values of $\bar{E}\%$ are 1.00 and 3.66 for prickly pear peels and prickly pear pomace, respectively. The obtained values of $\bar{E}\%$ are quite acceptable, indicating that the model proposed by Conte et al. [41], as applied to grape pomace, adequately describes the dehydration kinetics of the investigated by-products. The same dual-stage model adequately described the dehydration data of tomato peels and seeds [40]. In this last case, the influence of tomato by-product dehydration on temperature was also assessed by calculating the dehydration rate as a function of the by-product water content; the results demonstrated that the dehydration rate increased as the testing temperature increased. The values of the model parameters obtained by fitting the data in the current study are listed in Table 3.

**Figure 5:**
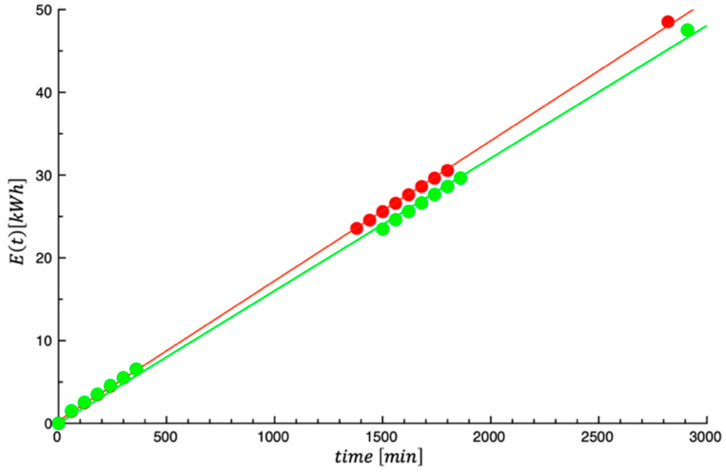
Energy consumption of the investigated prickly pear by-products. Green color = prickly pear pomace; red color = prickly pear peels.

Figure 5 shows the energy consumed by the dehydrator plotted as a function of time. The curves shown in the same figure are the best fit of Equation (8) to the data. The calculated values of $\bar{E}\%$ are 5.44 and 9.21 for prickly pear peels and prickly pear pomace, respectively. The $\bar{E}\%$ values obtained are not as good as those previously obtained for the dehydration process; however, they can be considered acceptable. The values of γ obtained by fitting the data are listed in Table 3.

**Figure 6:**
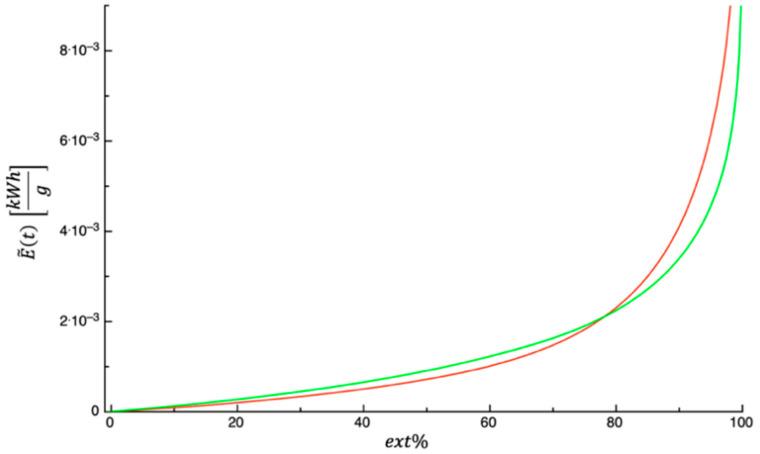
Energy consumption per gram of dehydrated matrix plotted as a function of ext%. Green color = prickly pear pomace; red color = prickly pear peels.

Figure 6 shows $\tilde{E}\left(ext\%\right)$ plotted as a function of ext%. The curves shown in the figure refer to the investigated by-products, which were predicted by means of Equations (10) and (11). The values of the parameters appearing in the above-mentioned equations are those listed in Table 3. The amount of energy consumed per gram of dehydrating by-product was calculated by setting the extent of the dehydration process to 99%; the obtained values are 0.010742 and 0.0070052 $\left[\frac{\mathrm{kWh}}{g}\right]$ for prickly pear peels and prickly pear pomace, respectively. The reader is invited to refer to the study of Panza et al. [63] to deepen their understanding of the aspects related to the analysis of energy consumption and how energy consumption can be optimized in the dehydration process.

### 3.3. Environmental Impact of Cheesecake with and Without Prickly Pears

**Figure 7:**
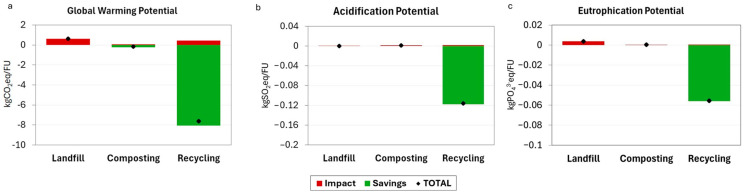
Impacts in kg of emissions per FU. The environmental impacts are represented in red, whereas the environmental savings are represented in green. The marker on each bar in the graph represents the total emissions calculated as the difference between the positive and negative values. (**a**) Global Warming Potential (kgCO_2_eq/FU), (**b**) Acidification Potential (kgSO_2_eq/FU), (**c**) Eutrophication Potential (kgPO_4_^3−^eq/FU). For all three impact categories, recycling is revealed as the most sustainable waste management option.

In this section, positive results represent environmental impacts (in red), whereas negative results represent environmental benefits (in green). Figure 7a shows that landfilling emerges as the largest contributor to GWP, with a value of 0.62 kgCO_2_eq/FU. This is consistent with the results from the review by Bernstad and Jansesn [64], in which landfilling was identified as the least favorable option among the various biowaste management scenarios analyzed. Indeed, European Union waste management regulations have established the obligation to reduce the amount of biodegradable organic waste sent to landfills (Directive 1999/31/EC; Directive EU 2018/850). The emissions from recycling processes record a value of 0.43 kgCO_2_eq/FU, which is influenced by the energy consumption required for dehydration and grinding operations. Finally, composting processes contributes in a lesser manner than that of landfill and recycling (0.06 kgCO_2_eq/FU).

In terms of environmental savings, recycling is the most advantageous solution, saving about 8.06 kgCO_2_eq/FU. This benefit arises from the reduced use of raw materials required to produce the fortified cheesecake compared to the traditional formulation. Composting also shows an environmental saving related to the application of compost to the soil (−0.23 kgCO_2_eq/FU). This process sequesters carbon from food waste, trapping it in the soil and reducing the CO_2_ that would otherwise be released into the atmosphere. Additionally, composting improves soil water retention and reduces the risk of erosion, preventing nutrient loss [65]. Considering both the impacts and environmental savings associated with the GWP category, recycling is the most sustainable process. According to Figure 7b, industrial composting and recycling exhibit similar low positive values (0.0012 and 0.0018 kgSO_2_eq/FU, respectively). This can be explained by the fact that composting generates decomposition emissions, which influences this impact category, as has also been highlighted by Zhang et al. [66]. Landfill presents a lower value than the other two options, which is therefore negligible. Recycling generates substantial environmental savings for this impact category (–0.118 kgSO_2_eq/FU) due to the reduction in raw material used to produce the fortified cheesecake. Considering both the burdens and environmental benefits, recycling emerges as the best biowaste management option for this impact category. In Figure 7c, landfill records the highest impact value (0.0036 kgPO_4_^3−^eq/FU). Composting and recycling, on the other hand, show similar and lower values (0.00029 and 0.00049 kgPO_4_^3−^eq/FU, respectively). These results are consistent with those of Keng et al. [67], who assessed the treatment of biowaste with composting and landfill in terms of Eutrophication Potential, obtaining a similar outcome. Recycling generates significant environmental savings (–0.056 kgPO_4_^3−^eq/FU) due to the reduced need for raw materials in the production of the fortified product and associated PO_4_^3−^eq emissions. When environmental impacts and savings are considered, recycling emerges as the most sustainable biowaste management option for this impact category.

**Figure 8:**
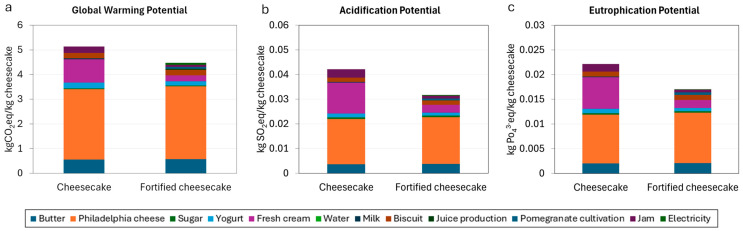
Impacts in kg of emissions per kg of cheesecake. Each ingredient and associated impacts are represented by a different color. (**a**) Global Warming Potential (kgCO_2_eq/kg cheesecake), (**b**) Acidification Potential (kgSO_2_eq/kg cheesecake), and (**c**) Eutrophication Potential (kgPO_4_^3−^eq/kg cheesecake). For all three impact categories, fortified cheesecake reduces the environmental impact.

For the second objective of the paper (Figure 8), we observe impacts per kg of cheesecake prepared, comparing the traditional cheesecake with the fortified cheesecake. In terms of GWP, 1 kg of fortified cheesecake reduces by 0.66 kg of CO_2_ eq with respect to 1 kg of traditional cheesecake (Figure 8a), primarily due to the reduction in the amount of ingredients required to produce the former. Indeed, comparing the two cheesecakes, the largest contribution to environmental savings is attributed to fresh cream (−0.70 kgCO_2_eq/kg cheesecake), followed by jam (−0.17 kgCO_2_eq/kg cheesecake). Other ingredients contributing to the environmental savings are plain yogurt (−0.07 kgCO_2_eq/kg cheesecake) and milk (−0.04 kgCO_2_eq/kg cheesecake). At the industrial level, this means that the substitution of the traditional cheesecake with a fortified cheesecake would correspond to a saving in relation to the raw materials used to obtain the final product. It should be noted that the results in Figure 8 are presented per kg of cheesecake, which has been assumed as a standardized weight for a single cheesecake unit. The environmental benefits shown per kg should therefore be extended to large-scale production when industrial activities adopt this solution.

The preparation of the fortified cheesecake also resulted in an overall environmental saving of 0.01 kg of SO_2_eq/kg cheesecake (Figure 8b) and 0.005 kg of PO_4_^3−^eq/kg cheesecake (Figure 8c). These outcomes can be attributed to the reduced quantities of the previously mentioned ingredients, which approximately follow the same order of impact as reported for the GWP. The results obtained from this study reveal that the recycling of biowaste, in this case prickly pear, represents an important option to reduce environmental impacts with respect to more traditional waste management systems. Replacing food items with recycled biowaste may result in a general reduction in emissions and resources used. Further analyses evaluating the social and economic aspects associated with the different management techniques of organic waste [68,69] are expected and could provide additional important insights to support studies like this. When, in the recycling process, livestock-derived products are replaced, the environmental benefits are significant due to the well-known environmental footprint associated with these food products [70,71]. For instance, in the case of cheesecake, part of the cow’s milk used in the traditional cheesecake is replaced by recycled biowaste. As confirmed in another study, dairy products are among the main drivers of the environmental impacts in relation to cheesecake preparation [72].

The results have shown how each environmental impact category analyzed contributes to the overall sustainability of the process. Indeed, reducing GWP implies a mitigation on global warming; reducing AP implies a mitigation of the acidification of soil and water; reducing EP implies a mitigation of the over-fertilization of water and soil.

Although the results presented are contextualized for a small-scale analysis, they may correlate to broader sustainability goals. Indeed, European Directives highlight the crucial role of recycling to address circular economy objectives. In this context, the transformation of biowaste into value-added products is recommended in line with a circular economy perspective. Indeed, in the Waste Framework Directive, the separate collection of organic waste and its relative treatment according to the waste hierarchy is strongly recommended in relation to the aim of supporting a circular economy transition.

## 4. Conclusions

The results of the current study contribute to the valorization of agro-industrial by-products, as the experimental evidence demonstrates that peels and pomace from prickly pears can be advantageously recycled to produce a new tasty cheesecake. In addition, the results highlight the fact that fortified cheesecake was also interesting from a compositional point of view, being richer in lipids, carbohydrates, and dietary fibers compared to the traditional dessert. Due to this increased compositional value, the fortified cheesecake recorded a total energetic value higher than that of the control product. By using a standardized LCA, the study shows that recycling always emerges as the most sustainable biowaste management option. The environmental benefits for recycling were observed in terms of three environmental impact categories. The current results can be linked to broader implications for sustainability and for the food industry, which is trying to redesign products and production methods to create healthier and more sustainable foods. The reduction in the amount of the five ingredients required to produce the fortified cheesecake compared to the traditional recipe, and of ingredients from the dairy sector, allowed us to demonstrate that replacing food items with recycled biowaste may result in a general increase in the environmental performance. From an academic point of view, further research is still necessary to widen the exploration of different by-product typologies and investigate their potential in developing more sustainable food for humans and the environment. It is also worth considering that research advances in by-product recycling could tackle one of the major food-related challenges facing modern society, only if industrial and government scientists, as well as other stakeholders such as consumers, policymakers and farmers, are involved in transforming the way we produce and consume foods. Concerning the sustainability analysis, this study focused on a single dimension of sustainability, i.e., the environmental one. Hence, future advancements including economic and social aspects linked to different treatments of biowaste and its subsequent introduction into the market are expected and recommended.

## Figures and Tables

**Table 1:** Ingredients used to formulate the control and the fortified cheesecakes.

	Ingredients	Control Cheesecake [g]	Fortified Cheesecake [g]
BASE	Biscuits	115	115
	Butter	50	50
FILLING	Philadelphia cheese	175	175
	Sugar	50	50
	Plain Yogurt	100	70
	Fresh cream	100	25
	Powdered sugar	10	10
	Isinglass	4	4
	Water	20	0
	Milk	20	0
	Prickly pear peel powder	0	25.77
	Prickly pear pomace powder	0	27.98
	Prickly pear juice	0	95
TOPPING	Jam	125	40
	Water	15	0
	Pectin	2	2
	Prickly pear peel powder	0	7.6
	Prickly pear pomace powder	0	8.3
	Prickly pear juice	0	55

**Table 2:** Centesimal composition of the control and the fortified cheesecake.

	Humidity [%]	Lipids [g/100 g]	Carbohydrates [g/100 g]	Proteins [g/100 g]	Total Fibers [g/100 g]	Sodium [mg/kg]	Sodium Chloride [mg/kg]	Ash [g/100 g]	Energetic Value [kcal/100 g]
Control cheesecake	51.36	12.94	26.26	4.56	4.2	0.14	0.35	0.68	248
Fortified cheesecake	44.9	16.98	27.38	3.98	5.7	0.14	0.34	1.24	290

**Table 3:** Values of the parameters used to estimate $\tilde{E}\left(99\%\right)$.

Parameters	Prickly Pear Peels	Prickly Pear Pomace
$t_{c}\;\left[\min\right]$	158.4938	299.8881
$k_{1}\left[\frac{g\;\mathrm{desorbed}\;\mathrm{water}}{100\;g\;\mathrm{dry}\;\mathrm{matter}}\cdot \frac{1}{\min}\right]$	1.5326	0.6584
$k_{2}\;\left[\frac{g\;\mathrm{desorbed}\;\mathrm{water}}{100\;g\;\mathrm{dry}\;\mathrm{matter}}\right]$	210.229	92.2308
$\gamma \;\left[\frac{\mathrm{kWh}}{\min}\right]$	0.0171	0.016019
$x_{dm}^{0}$	0.182410614	0.244418642
$m_{Tot}^{0}\;\left[g\right]$	6000	6000

## Data Availability

The original contributions presented in the study are included in the article, further inquiries can be directed to the corresponding author.

## Supplementary Materials

The following supporting information can be downloaded at: https://www.mdpi.com/article/10.3390/foods14071159/s1. Figure S1: sensory evaluation form; Table S1: inventory data related to the three biowaste management options; Table S2: inventory data related to the ingredients used in both cheesecakes.
