# Supplementary material for: Cheesecake Customized Using Juice and By-Products from Prickly Pears: A Case Study of Recycling and Environmental Impact Evaluation
Source: Foods. 2025 Mar 26;14(7):1159. doi: 10.3390/foods14071159 (PMC11988937; doi:10.3390/foods14071159)
Supplement: Supplementary file 1 [file foods-14-01159-s001.zip › foods-3529246-supplementary.pdf]

Supporting Data for the Article

Cheesecake customized using both juice and by-products from prickly pears: a case study of recycling and environmental impact evaluation

Sensory evaluation form

Directions: Check one rating for each of the following: texture, flavour, color, taste and overall acceptability for both cheesecake types, considering the base, the filling and the topping.

Product code:

Rating scale	Colour	Texture	Flavour	Taste	Overall acceptability
9. Like extremely					
8. Like very much					
7. Like moderately					
6. Like slightly					
5. Neither like or dislike					
4. Dislike slightly					
3. Dislike moderately					
2. Dislike very much					
1.Dislike extremely					
Comments and suggestions:					

Product code:

Rating scale	Colour	Texture	Flavour	Taste	Overall acceptability
9. Like extremely					
8. Like very much					
7. Like moderately					
6. Like slightly					
5. Neither like or dislike					
4. Dislike slightly					
3. Dislike moderately					
2. Dislike very much					
1.Dislike extremely					
Comments and suggestions:					

Panelist code: Gender and age: Date:

Table S1. The Table presents the inventory data related to the three biowaste management options. The values are referred to the functional unit.

Process	Value	Unit	Source	Name of the data used in the database	Reference
Composting	1	kg		Biowaste {RoW} treatment of biowaste, industrial composting |Cut-off, S	Ecoinvent (2024)
Composting (Savings)	1	kg			Saer et al. 2013
Landfill	1	kg		Biowaste {RoW} treatment of biowaste, open dump |Cut-off, S	Ecoinvent (2024)
Recycling	2,006	kWh	University of Foggia	Electricity, medium voltage {IT}|market off | Cut-off, S	Ecoinvent (2024)

Table S2. The table presents the inventory data related to each ingredient used in the preparation of both the traditional (TC) and fortified (FC) cheesecakes. The values are referred to the functional unit.

	Ingredient	Value TC	Value FC	Unit	Source	Name of data used in the database	Reference
Crust	Biscuit	1,597	1,65	kg	University of Foggia		Environdec (2020)
	Butter	0,694	0,71	kg	University of Foggia	Butter, from cow milk {GLO}|production| Cut-off, S	Ecoinvent (2024)
	Peel powder	0	0	kg	University of Foggia		
	Pomace powder	0	0	kg	University of Foggia		
Filling	Philadelphia Cheese	2,43	2,51	kg	University of Foggia	Cheese, from cow milk, fresh, unripened {GLO}| cheese production, soft, from cow milk |Cut-off, S	Ecoinvent (2024)
	Sugar	0,69	0,71	kg	University of Foggia	Sugar, from sugar beet {RoW}| beet sugar production | Cut-off, S	Ecoinvent (2024)
	Plain yogurt	1,38	1,00	kg	University of Foggia	Yogurt, from cow milk {RoW}|production| Cut-off, S	Ecoinvent (2024)
	Fresh Cream	1,38	0,35	kg	University of Foggia	Cream, full, from processing, at plant/NL Mass	Blonk Sustainability (2022)

	Powdered sugar	0,13	0,14	kg	University of Foggia	Sugar, from sugar beet {RoW}| beet sugar production | Cut-off, S	Ecoinvent (2024)
	Isinglass	0,05	0,05	kg	University of Foggia		
	Water	0,27	0	kg	University of Foggia	Tap water {Europe without Switzerland}| tap water production, conventional treatment | Cut-off, S	Ecoinvent (2024)
	Milk	0,27	0	kg	University of Foggia	Cow milk {RoW}| milk production, from cow | Cut-off, S	Ecoinvent (2024)
	Peel powder	0	0,36	kg	University of Foggia		
	Pomace powder	0	0,40	kg	University of Foggia		
	Juice	0	1,36	kg	University of Foggia		Environdec (2020)
Topping	Jam	1,73	0,57	kg	University of Foggia		Gallo et al., 2024
	Water	0,20	0	kg	University of Foggia	Tap water {Europe without Switzerland}| tap water production, conventional treatment | Cut-off, S	Ecoinvent (2024)
	Pectin	0,02	0,02	kg	University of Foggia		
	Peel powder	0	0,10	kg	University of Foggia		
	Pomace powder	0	0,11	kg	University of Foggia		

	Juice	0	0,79	kg	University of Foggia		Environdec (2020)

References

Blonk Sustainability, 2022. Agri-footprint: Methodology Report.

Ecoinvent, 2024. Ecoinvent database version 3.

Environdec, 2020. Environmental Product Declaration: Pavesi Petit dried biscuit. https://api.environdec.com/api/v1/EPDLibrary/Files/be18f17e-9a6d-4260-a5f3-ea0920bb8a96/Data

Environdec, 2024. *Environmental Product Declaration: Cloudy Apple Juice NFC.* https://api.environdec.com/api/v1/EPDLibrary/Files/d456f870-eb2e-416c-96ff-08dc3c72202b/Data

Gallo, F., Manzardo, A., Camana, D., Fedele, A., & Scipioni, A. (2024). Integration of a circular economy metric with life cycle assessment: methodological proposal of compared agri-food products. The International Journal of Life Cycle Assessment, 29(8), 1359-1379.

Saer, A., Lansing, S., Davitt, N. H., & Graves, R. E. 2013. Life cycle assessment of a food waste composting system: environmental impact hotspots. Journal of Cleaner Production, 52, 234-244.
